# A robust ECC based mutual authentication protocol with anonymity for session initiation protocol

**DOI:** 10.1371/journal.pone.0186044

**Published:** 2017-10-20

**Authors:** Zahid Mehmood, Gongliang Chen, Jianhua Li, Linsen Li, Bander Alzahrani

**Affiliations:** 1 School of Electronic Information and Electrical Engineering, Shanghai Jiao Tong University, Shanghai, China; 2 Faculty of Computing and Information Technology, King Abdulaziz University, Jeddah, Saudia Arabia; King Saud University, SAUDI ARABIA

## Abstract

Over the past few years, Session Initiation Protocol (SIP) is found as a substantial application-layer protocol for the multimedia services. It is extensively used for managing, altering, terminating and distributing the multimedia sessions. Authentication plays a pivotal role in SIP environment. Currently, Lu et al. presented an authentication protocol for SIP and profess that newly proposed protocol is protected against all the familiar attacks. However, the detailed analysis describes that the Lu et al.’s protocol is exposed against server masquerading attack and user’s masquerading attack. Moreover, it also fails to protect the user’s identity as well as it possesses incorrect login and authentication phase. In order to establish a suitable and efficient protocol, having ability to overcome all these discrepancies, a robust ECC-based novel mutual authentication mechanism with anonymity for SIP is presented in this manuscript. **The improved protocol contains an explicit parameter for user to cope the issues of security and correctness** and is found to be more secure and relatively effective to protect the user’s privacy, user’s masquerading and server masquerading as it is verified through the comprehensive formal and informal security analysis.

## 1 Introduction

The applications of multimedia services have great significance in advanced networks. The SIP is a valued application-layer protocol used in controlling and signaling the multimedia sessions. The prime responsibility of SIP is the internet telephone services such as voice call, video call and instant messaging over the public network. Furthermore, SIP is responsible to establish, modify and terminate the multimedia sessions [1]. Authentication process is performed by the users in order to login the server through SIP. So, the authentication plays a vital role for the SIP protocol services. Nowadays, due to keen interest of the researchers for security maintenance and authentication of SIP, there is an immense scope for research in authentication of multimedia services. Recently, numerous scholars have presented some secure and efficient authentication techniques to sustain the security of SIP [2–6]. Many researchers have consensus that Hypertext Transport Protocol (HTTP) digest authentication for SIP is found vulnerable for stolen verifier, server spoofing and off-line password guessing attacks and unable to provide mutual authentication [1, 7–9]. In order to counter these weaknesses, Yang et al. [7] introduced an authentication protocol based on Diffie-Hellman key exchange protocol. Afterwords, Huang et al. [10] pointed out that Yang et al.’s protocol fails to resist off-line password guessing attack and proposed an updated scheme to fix the identified issues appeared in Yang et al.’s scheme. However, Huang’s protocol is found unprotected against off-linen password guessing attack indicated by Jo et al. [11]. In order to enhance Yang et al.’s proposed technique, Durlanik and Sogukpinar [12] presented a secure as well as effective authentication technique based on Elliptic Curve Cryptography (ECC) [13]. ECC can provide same security with relatively smaller key size than the other cryptosystems [13–18]. In 2009, Wu et al. [19] presented an improved and secure authentication protocol for SIP based on ECC. Later on, Yoon et al. [20] demonstrated that Durlanik and Sogukpinar as well as Wu et al.’s proposed protocols are not secure against Denning-Sacco [21], off-line password guessing and stolen verifier attacks. Then Yoon et al.’s introduced a sophisticated technique for SIP with higher security. Pu et al. [22] identified that Yoon et al.’s technique is vulnerable against replay and off-line password guessing attacks. Then a comparatively lightweight authentication and a key agreement protocol by using hash function and exclusive-OR operation is presented by Tsai [23]. Later on, Arshad and Ikram [24] proved that Tsai’s protocol is breakable against the off-line password guessing and stolen-verifier attacks. Moreover, Tsai’s protocol remained unable to maintain forward secrecy and known-key secrecy. Though Yoon et al. [25] presented a robust authentication technique with a key agreement to address the limitations of Tsai’s scheme, yet Yoon et al.’s scheme is found unprotected against off-line password guessing and stolen verifier attacks indicated by Xie [26] and introduced a new scheme. Unfortunately, Xie’s protocol is exposed against off-line password guessing and impersonation attacks indicated by Farash and Attari [27]. Moreover, they proposed a new technique to counter the limitations of Xie’s scheme. Zhang et al. [28] offered an authentication protocol by using ECC with anonymity. Recently, Lu et al. [29] indicated that Zhang et al.’s scheme was breakable in case of insider attack and failed to offer mutual authentication. To remedy these vulnerabilities, Lu et al.’s [29] suggested an advance scheme, which is claimed to be more appropriate against all possible attacks. However, it is analyzed that Lu et al.’s proposed protocol is found insecure in case of server and user masquerading attacks. Additionally, it also fails to offer user anonymity accompanied with incorrect scheme. Hence, a new robust mutual authentication protocol with anonymity using ECC for SIP is presented in this manuscript. **The improved scheme contains a slight modification in both registration and authentication phases. We have supplemented an explicit parameter for user to cope the issues of correctness and security**. Furthermore, the protocol is highly secured against all the possible attacks as validated through informal and formal security analysis. Comprehensive analysis also verifies that security and performance of the proposed protocol is more effective and reliable as compared to the recent authentication protocols.

The remainder of the manuscript is arranged as follows: Table 1 describes the notations used in this manuscript. Review and weaknesses of Lu et al.’s scheme is demonstrated in sections 2 and 3, respectively. The proposed protocol is presented in section 4, formal and informal security are figured out in section 5. Performance comparison of the present protocol with recently presented protocols is investigated in sections 6. Concluding statement of the proposed protocol is elaborated in section 7.

**Table 1:** Notation guide.

Notations	Description
$S,U_{i}$	Server and User
*ID*_*i*_, *PW*_*i*_	User Identity and Password
*r*_1_, *r*_2_	Two Random Numbers
$P_{ui},P_{s}$	User and Server Private Key
*h*(.)	One-way hash functions
‖	Concatenation operation
⊕	XOR operation
*E*_*p*_(*a*, *b*)	Elliptic Curve over *F*_*p*_
*P*	Value of Elliptic Curve Point
A	The Adversary

## 2 Review of Lu et al.’s scheme

**Fig 1:**
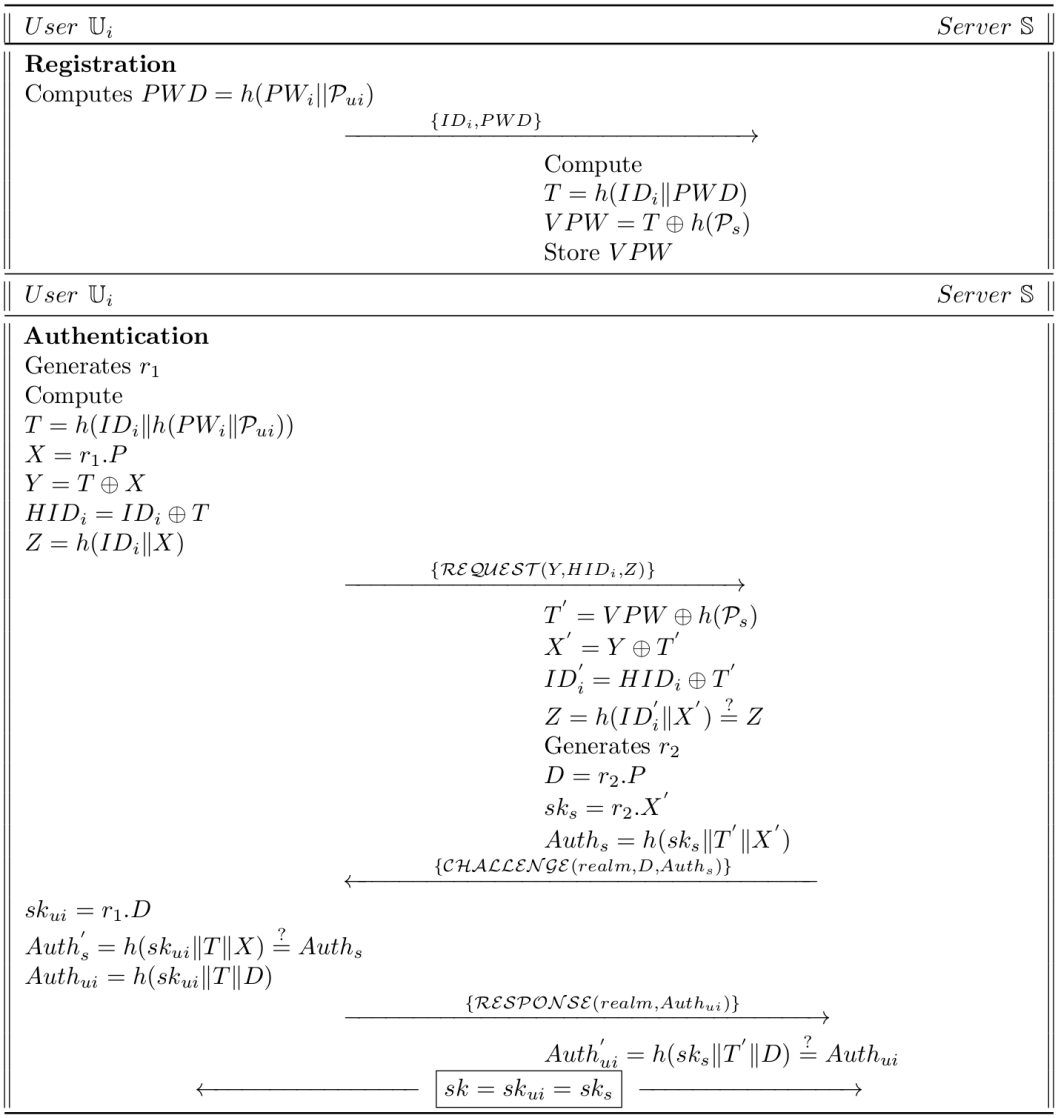
Lu et al.’s scheme.

This segment concisely demonstrates the detail analysis of Lu et al.’s protocol. The overall protocol contains three stages. At the first stage, the user performs the registration process, then use it to login into the server and authenticate itself. It also permits the user to update his/her password in inadmissible condition. All these phases are explained in detail and Fig 1 shows the registration and authentication Phases as follows:

### 2.1 Registration phase

1. $U_{i}$ chooses his/her *ID*_*i*_, *PW*_*i*_ and his/her secret key $P_{ui}$. $U_{i}$ now computes $PWD=h(PW_{i}\|P_{ui})$ and transmits {*ID*_*i*_, *PWD*} to S by private channel.
2. S determines $V\;PW=h\left(ID_{i}\|PWD\right)⊕h\left(P_{s}\right)$ upon receiving the message and stores it in server’s repository.

### 2.2 Authentication phase

1. A random number *r*_1_ is generated and computed by $U_{i}$:
  $$\begin{array}{cc} & T=h(ID_{i}\|h\left(PW_{i}\|P_{ui}\right)) \end{array}$$ (1)
  $$\begin{array}{cc} & X=r_{1}.P \end{array}$$ (2)
  $$\begin{array}{cc} & Y=T⊕X \end{array}$$ (3)
  $$\begin{array}{cc} & HID_{i}=ID_{i}⊕T \end{array}$$ (4)
  $$\begin{array}{cc} & Z=h(ID_{i}\|X) \end{array}$$ (5)
  Now the $U_{i}$ transmits the $REQUEST\{Y,HID_{i},Z\}$ to S.
2. S computes, upon reception of request message from $U_{i}$:
  $$\begin{array}{cc} & T^{{}^{\prime }}=V\;PW⊕h\left(P_{s}\right) \end{array}$$ (6)
  $$\begin{array}{cc} & X^{{}^{\prime }}=Y⊕T^{{}^{\prime }} \end{array}$$ (7)
  $$\begin{array}{cc} & ID_{i}^{{}^{\prime }}=HID_{i}⊕T^{{}^{\prime }} \end{array}$$ (8)
  $$\begin{array}{cc} & Z^{{}^{\prime }}=h\left(ID_{i}^{{}^{\prime }}\|X^{{}^{\prime }}\right) \end{array}$$ (9)
  verify $Z^{{}^{\prime }}\overset{?}{=}Z$, in case of failure, the session is aborted otherwise, a random number *r*_2_ is generated by S and calculates:
  $$\begin{array}{cc} & D=r_{2}.P \end{array}$$ (10)
  $$\begin{array}{cc} & sk_{s}=r_{2}.X^{{}^{\prime }} \end{array}$$ (11)
  $$\begin{array}{cc} & Auth_{s}=h(sk_{s}\|T^{{}^{\prime }}\left\|X^{{}^{\prime }}\right) \end{array}$$ (12)
  Now S sends the challenge message $CHALLENGE\{realm,D,Auth_{s}\}$ to user $U_{i}$.
3. $U_{i}$ on getting the challenge message from S calculates:
  $$\begin{array}{cc} & sk_{ui}=r_{1}.D \end{array}$$ (13)
  checks $Auth_{s}^{{}^{\prime }}\overset{?}{=}h(sk_{ui}\left\|T\|X\right)$ is equal to received *Auth*_*s*_, if the equation is not satisfied, $U_{i}$ aborts the session, otherwise $U_{i}$ computes *Auth*_*ui*_ = *h*(*sk*_*ui*_‖*T*‖*D*) and transmits the $RESPONSE\{realm,Auth_{ui}\}$ message to S.
4. Upon getting the message, S verifies $Auth_{ui}^{{}^{\prime }}=h(sk_{s}\|T^{{}^{\prime }}\left\|D\right)\overset{?}{=}Auth_{ui}$, if it true, the already computed session key *sk* = *sk*_*ui*_ = *sk*_*s*_ is treated as legitimate key.

### 2.3 Password change phase

$U_{i}$ selects new $PW_{i}^{new}$, $P_{ui}^{new}$ and $r_{1}^{new}$ in order to change the password.

The following strides are performed by the $U_{i}$ and S.

1. $U_{i}$ computes:
  $$\begin{array}{cc} & W=h(sk\|h\left(ID_{i}\|h\left(PW_{i}\|P_{ui}\right)\right)) \end{array}$$ (14)
  $$\begin{array}{cc} & N=h\left(ID_{i}\|sk\right)⊕h\left(ID_{i}\|h\left(PW_{i}^{new}\|P_{ui}^{new}\right)\right) \end{array}$$ (15)
  Now $U_{i}$ transmits {*ID*_*i*_, *W*, *N*} to the S
2. On getting the message, S concludes: $W^{{}^{\prime }}=h\left(sk\|VPW⊕h\left(P_{s}\right)\right)$ and verifies whether it is equal to the acquired *W*. Then S computes $V\;PW^{new}=h\left(P_{s}\right)⊕h_{1}\left(ID_{i}\|sk\right)⊕N$ and updates *V PW* with *V PW*^*new*^.

## 3 Cryptanalysis of Lu et al.’s scheme

In this section, it is revealed that the Lu et al.’s scheme is impressionable to server and user masquerading attacks and also unable to achieve the user anonymity. Moreover, it also has incorrect authentication phase on server side. As per adversary model mentioned in [30–34], A can access the public communication link and can replay, remove, modify, intercept or can send a new devised message.

### 3.1 User anonymity attack

The user anonymity violation is observed in this subsection. In the Lu et al.’s protocol, any legitimate user can derive the authentic identity of the specific user by intercepting the login request message from the public communication channel.

Assume a legal user $U_{j}$ try to extract the authentic identity of the another user $U_{i}$. $U_{j}$, performs the following steps.

1. User $U_{j}$ steals the information *V PW* stored on the server.
2. Now by using his/her *ID*_*j*_, *PW*_*j*_ and $P_{uj}$, $U_{j}$ computes $PWD_{j}=h\left(PW_{j}\|P_{uj}\right)$ and obtains the value *T* = *h*(*ID*_*j*_‖*PWD*_*j*_). Now, $U_{j}$ can also extract the server private key $h(P_{s})=T⊕V\;PW$.
3. $U_{j}$ intercepts the $U_{i}$ login request message {*Y*, *HID*_*i*_, *Z*} and sends it to the server S from the public communication channel.
4. Now by using the stolen verifier *V PW*, he/she can compute $T^{{}^{\prime }}=V\;PW⊕h\left(P_{s}\right)$. Finally, $U_{j}$ extracts the real identity *ID*_*i*_ of user’s $U_{i}$ as $ID_{i}^{{}^{\prime }}=HID_{i}⊕T^{{}^{\prime }}$.

### 3.2 Server masquerading attack

In Lu et al.’s scheme, if A devised the server S’s secret key $P_{s}$, another authorized user $U_{j}$ can easily masquerade as a legal server by executing the subsequent steps.

1. Adversary A can steal the information *V PW* stored in the server’s repository. Then the following steps have to be performed by the A
  $$\begin{array}{cc} & PWD_{j}=h\left(PW_{j}\|P_{uj}\right) \end{array}$$ (16)
  $$\begin{array}{cc} & T=h(ID_{j}\|PWD_{j}) \end{array}$$ (17)
  $$\begin{array}{cc} & h(P_{s})=T⊕V\;PW \end{array}$$ (18)
2. When $U_{i}$ a legal user needs to login into the server,$U_{i}$ calculates:
  $$\begin{array}{cc} & generates\;r_{1} \end{array}$$ (19)
  $$\begin{array}{cc} & T=h(ID_{i}\|h\left(PWi\|P_{ui}\right)) \end{array}$$ (20)
  $$\begin{array}{cc} & X=r_{1}.P \end{array}$$ (21)
  $$\begin{array}{cc} & Y=T⊕X \end{array}$$ (22)
  $$\begin{array}{cc} & HID_{i}=ID_{i}⊕T \end{array}$$ (23)
  $$\begin{array}{cc} & Z=h(ID_{i}\|X) \end{array}$$ (24)
  Now $U_{i}$ conveys the request message {*Y*, *HID*_*i*_, *Z*} to the server S.
3. A computes after intercepting the request message:
  $$\begin{array}{cc} & {\bar{T}}^{{}^{\prime }}=V\;PW⊕h\left(P_{s}\right) \end{array}$$ (25)
  $$\begin{array}{cc} & {\bar{X}}^{{}^{\prime }}=\bar{Y}⊕{\bar{T}}^{{}^{\prime }} \end{array}$$ (26)
  $$\begin{array}{cc} & {\bar{ID_{i}}}^{{}^{\prime }}=\bar{HID_{i}}⊕{\bar{T}}^{{}^{\prime }} \end{array}$$ (27)
  $$\begin{array}{cc} & generates\;r_{2} \end{array}$$ (28)
  $$\begin{array}{cc} & D=r_{2}.P \end{array}$$ (29)
  $$\begin{array}{cc} & sk_{s}=r_{2}.{\bar{X}}^{{}^{\prime }} \end{array}$$ (30)
  $$\begin{array}{cc} & Auth_{s}=h(sk_{s}\|{\bar{T}}^{{}^{\prime }}\left\|{\bar{X}}^{{}^{\prime }}\right) \end{array}$$ (31)
  After that A transmits $CHALLANGE(realm,D,Auth_{s})$ to the legal user $U_{i}$
4. On getting the challenge message, $U_{i}$ computes:
  $$\begin{array}{cc} & sk_{ui}=r_{1}.D \end{array}$$ (32)
  $$\begin{array}{cc} & Auth_{s}^{{}^{\prime }}=h(sk_{ui}\left\|T\|X\right)=Auth_{s} \end{array}$$ (33)
  $$\begin{array}{cc} & Auth_{ui}=h(sk_{ui}\left\|T\|D\right) \end{array}$$ (34)
5. The $U_{i}$ conveys the response message $RESPONSE(realm,Auth_{ui})$ to the server S, whereas, A intercepts the message. Hence, A successfully masquerades the server for legal users.

### 3.3 User masquerading attack

For user masquerading attack, an attacker A will get the request message {*Y*, *HID*_*i*_, *Z*} and derive the identity *ID*_*i*_ as mentioned in 3.1. Now, any legitimate user $U_{j}$ can easily masquerade another user $U_{i}$ by performing subsequent steps:

1. A intercepts the remote user $U_{i}$ request message {*Y*, *HID*_*i*_, *Z*} and computes:
  $$\begin{array}{cc} & {\bar{T}}^{{}^{\prime }}=HID_{i}⊕ID_{i} \end{array}$$ (35)
  $$\begin{array}{c} Generates\;r_{1} \end{array}$$ (36)
  $$\begin{array}{cc} & {\bar{X}}^{{}^{\prime }}=r_{1}.P \end{array}$$ (37)
  $$\begin{array}{cc} & \bar{Y}=\bar{T}⊕{\bar{X}}^{{}^{\prime }} \end{array}$$ (38)
  $$\begin{array}{cc} & \bar{Z}=h\left(ID_{i}\|{\bar{X}}^{{}^{\prime }}\right) \end{array}$$ (39)
A transmits his own request message $\left\{\bar{Y},\bar{HID_{i}},\bar{Z}\right\}$.
2. S upon getting the message computes:
  $$\begin{array}{cc} & T^{{}^{\prime }}=V\;PW⊕h\left(P_{s}\right) \end{array}$$ (40)
  $$\begin{array}{cc} & X^{{}^{\prime }}=\bar{Y}⊕T^{{}^{\prime }} \end{array}$$ (41)
  $$\begin{array}{cc} & ID_{i}^{{}^{\prime }}=\bar{HID_{i}}⊕T^{{}^{\prime }} \end{array}$$ (42)
  $$\begin{array}{c} verify\;Z=h\left(ID_{i}^{{}^{\prime }}\|X^{{}^{\prime }}\right)\overset{?}{=}Z \end{array}$$ (43)
  $$\begin{array}{c} Generates\;r_{2} \end{array}$$ (44)
  $$\begin{array}{c} Computes\;D=r_{2}.P \end{array}$$ (45)
  $$\begin{array}{cc} & sk_{s}=r_{2}.X^{{}^{\prime }} \end{array}$$ (46)
  $$\begin{array}{cc} & Auth_{s}=h(sk_{s}\|T^{{}^{\prime }}\left\|X^{{}^{\prime }}\right) \end{array}$$ (47)
S transmits the challenge message {*realm*, *D*, *Auth*_*s*_} to U
3. Upon getting the message A computes:
  $$\begin{array}{cc} & sk_{\bar{ui}}=r_{1}.D \end{array}$$ (48)
  $$\begin{array}{cc} & Auth_{ui}=h(sk_{\bar{ui}}\|{\bar{T}}^{{}^{\prime }}\left\|{\bar{X}}^{{}^{\prime }}\right)\overset{?}{=}Auth_{s} \end{array}$$ (49)
  $$\begin{array}{cc} & Auth_{ui}=h(sk_{\bar{ui}}\|{\bar{T}}^{{}^{\prime }}\left\|D\right) \end{array}$$ (50)
  Therefore, A sends the response message to S
4. On receiving the message, the S authenticates the adversary as a legal user by verifying *Auth*_*ui*_ = *h*(*sk*_*s*_‖*T*′‖*D*) equation. Therefore, A has successfully misled the server S and S treats the shared session key as a valid key.

### 3.4 Incorrectness problem

In this segment, it is demonstrated that while authentication is performed on the server side the server, computes $T^{{}^{\prime }}=VPW⊕h\left(P_{s}\right)$, where, $h(P_{s})$ is the private key of the server which is secret within the server. But how the server can compute the exact value of *T*’, without knowing the valid *ID*_*i*_ of the specific user as the verifier table contains the *T* values of all the legal users of the system.

## 4 Proposed scheme

**Fig 2:**
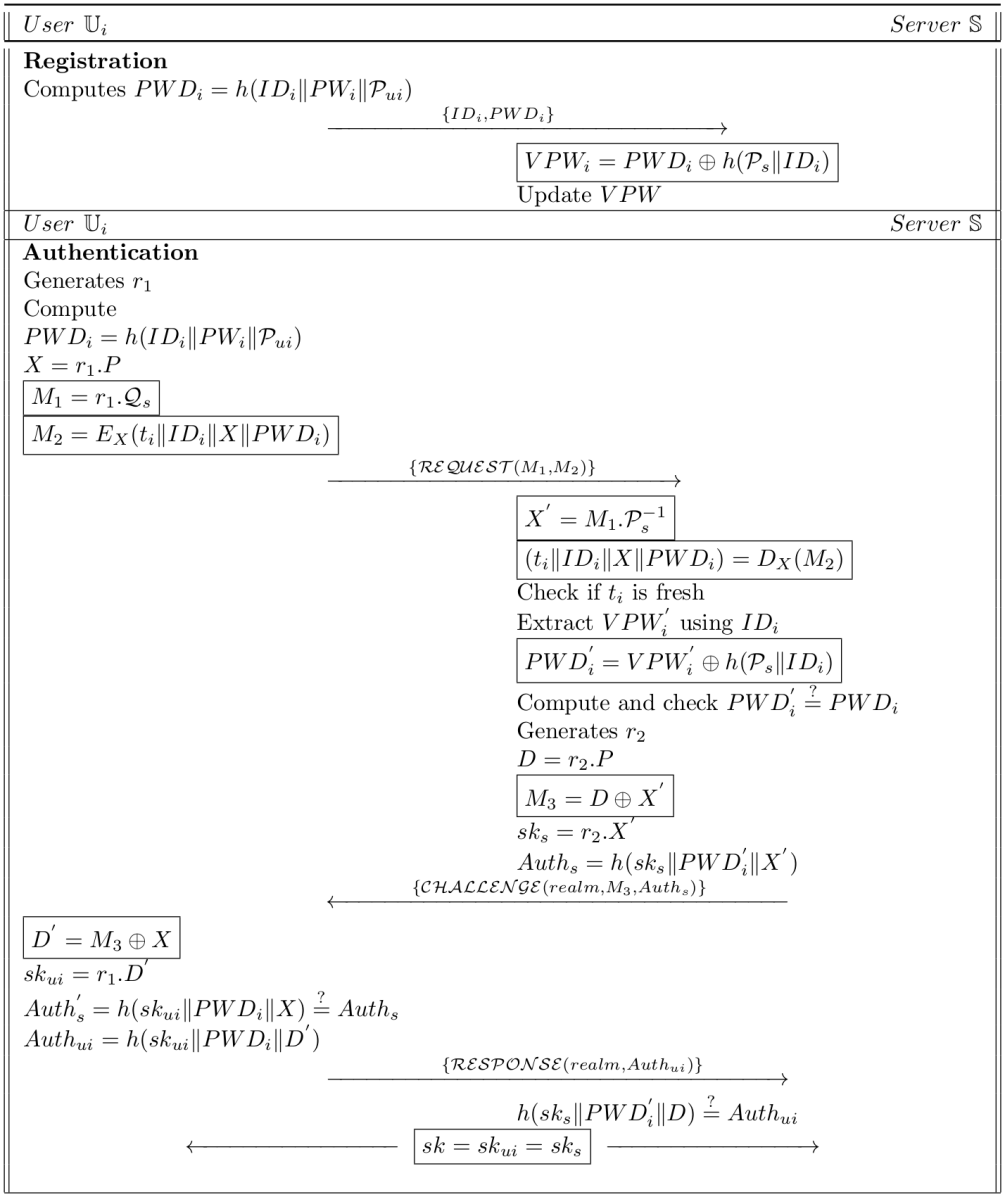
Proposed scheme.

In this segment, a new proposed protocol is presented. The improved protocol is divided into three phases, i.e., System Setup, Registration and Authentication phase. Before going into the details of proposed protocol, it explains that the insecurity of Lu et al.’s scheme against server and user’s masquerading attacks was due to a generic secret value $h(P_{s})$ hideously stored in the verifier table *V PW* into the server. A legitimate but dishonest user (say $U_{j}$) can easily extract $h(P_{s})$ by using his/her *T* which is computed as *T* = *h*(*ID*_*j*_‖*PWD*_*j*_) and then with the help of this *T*, it can compute server private key $h(P_{s})=T⊕V\;PW$. After obtaining $h(P_{s})$, the illegitimate but authorized user $U_{j}$ can easily find the real identity of any other user. Moreover, the $U_{j}$ after stealing the *V PW* from the server can easily masquerade himself as $U_{i}$ as well as the legal server. In present protocol, *V PW* consist of user’s particular identity *ID*_*i*_. Hence, if A successfully gets the secrets from the verifiers table, one can retrieve his/her own value of *PWD* as user *ID*_*i*_ is inserted with server secret key. So, he cannot masquerade himself as another user or server of the systems though he has also *V PW* verifier table. The scheme is illustrated in Fig 2 and is explain as follows:

### 4.1 System setup phase

1. The server S selects elliptic curve [35] points (*Ep*(*a*, *b*)) of order n and gets initialized with a base point *P*.
2. The secret key $P_{s}\in_{R}\to Z_{p}^{*}$ is being generated by S ranging from $\left(P_{s}\in \left[0,n-1\right]\right)$ and S computes the public key as $Q_{s}=P_{s}.P$. Then S selects one-way hash function *h*(), which keeps the secret key $P_{s}$ safe and publishes the rest of the public parameters.

### 4.2 Registration phase

1. $U_{i}$ computes $PWD_{i}=h(ID_{i}\|PW_{i}\left\|P_{ui}\right)$ and conveys {*ID*_*i*_, *PWD*_*i*_} to S through secure channel.
2. On getting the message, S computes $V\;PW_{i}=PWD_{i}⊕h\left(P_{s}\|ID_{i}\right)$ and saves the *V PW* in the server database.

### 4.3 Authentication phase

1. First of all, a random number *r*_1_ is generated by $U_{i}$ and it computes:
  $$\begin{array}{cc} & PWD_{i}=h(ID_{i}\|PW_{i}\left\|P_{ui}\right) \end{array}$$ (51)
  $$\begin{array}{cc} & X=r_{1}.P \end{array}$$ (52)
  $$\begin{array}{cc} & M_{1}=r_{1}.Q_{s} \end{array}$$ (53)
  $$\begin{array}{cc} & M_{2}=E_{X}(t_{i}\|ID_{i}\|X\|PWD_{i}) \end{array}$$ (54)
  Now $U_{i}$ sends request message $REQUEST\{M_{1},M_{2}\}$ to S.
2. S compute the following, upon getting the request message
  $$\begin{array}{cc} & X^{{}^{\prime }}=M_{1}.P_{s}^{-1} \end{array}$$ (55)
  $$\begin{array}{cc} & (t_{i}\|ID_{i}\|X\|PWD_{i})=D_{X}\left(M_{2}\right) \end{array}$$ (56)
  $$\begin{array}{cc} & Check\;if\;t_{i}\;isfresh \end{array}$$ (57)
  $$\begin{array}{cc} & Extract\;V\;PW_{i}^{{}^{\prime }}\;using\;ID_{i} \end{array}$$ (58)
  $$\begin{array}{cc} & PWD_{i}^{{}^{\prime }}=V\;PW_{i}^{{}^{\prime }}⊕h\left(P_{s}\|ID_{i}\right) \end{array}$$ (59)
  Compute and check $PWD_{i}^{{}^{\prime }}\overset{?}{=}PWD_{i}$, failure of which leads to the termination of session, otherwise S generates *r*_2_ and computes:
  $$\begin{array}{cc} & D=r_{2}.P \end{array}$$ (60)
  $$\begin{array}{cc} & M_{3}=D⊕X^{{}^{\prime }} \end{array}$$ (61)
  $$\begin{array}{cc} & sk_{s}=r_{2}.X^{{}^{\prime }} \end{array}$$ (62)
  $$\begin{array}{cc} & Auth_{s}=h(sk_{s}\|PWD_{i}^{{}^{\prime }}\left\|X^{{}^{\prime }}\right) \end{array}$$ (63)
S transmits the message {*realm*, *M*_3_, *Auth*_*s*_} to the $U_{i}$.
3. Upon receiving the message, $U_{i}$ calculates:
  $$\begin{array}{cc} & D^{{}^{\prime }}=M_{3}⊕X \end{array}$$ (64)
  $$\begin{array}{cc} & sk_{ui}=r_{1}.D^{{}^{\prime }} \end{array}$$ (65)
  and verifies the condition $Auth_{s}^{{}^{\prime }}=h(sk_{ui}\|PWD_{i}\left\|X\right)\overset{?}{=}Auth_{s}$, failure of which leads to the termination of the session, otherwise $U_{i}$ computes *Auth*_*ui*_ = *h*(*sk*_*ui*_‖*PWD*_*i*_‖*D*′) and transmits $RESPONSE(realm,Auth_{ui})$ message to S.
4. On receipt of the message {*realm*, *Auth*_*ui*_}, the S verifies $h(sk_{s}\|PWD_{i}^{{}^{\prime }}\left\|D\right)\overset{?}{=}Auth_{ui}$, if it withstands, the session key *sk* = *sk*_*ui*_ = *sk*_*s*_ is considered to be the valid key between $U_{i}$ and S.

## 5 Security analysis

Security analysis informal and formal of the present stated scheme demonstrates that it is resilient against all known attacks over public communication channels.

### 5.1 Informal security analysis

#### 5.1.1 Resist replay attack

Suppose if an eavesdropper can steal the request message {*M*_1_, *M*_2_} and try to replay it to pretend as a legal user $U_{i}$, but on the server side S verifies the freshness of time stamp *t*_*i*_ and also the condition $PWD_{i}^{{}^{\prime }}=V\;PW⊕h\left(P_{s}\|ID_{i}\right)\overset{?}{=}PWD_{i}$. To successfully pass the condition. A requires *ID*_*i*_ and server secret key $P_{s}$. But A is unable to get *ID*_*i*_ and server secret key $P_{ui}$ as they are secured by the One-way hash function. Furthermore, if A is able to get the challenge message {*realm*, *M*_3_, *Auth*_*s*_} from S and tries to replay it to $U_{i}$. A fails to obtain *r*_2_ from *D* and *Auth*_*s*_ is not equal to the computed *h*(*sk*_*ui*_‖*PWD*_*i*_‖*X*) by $U_{i}$. Then $U_{i}$ is failed to send response message to A. Hence, the present scheme is protected against replay attack.

#### 5.1.2 Anonymity and privacy

In the proposed scheme, the *ID*_*i*_ is protected on the public channel by hash function along with user secret key $P_{ui}$. So, it is impossible for A to get the *ID*_*i*_ from the public channel. Hence, the proposed scheme provides appropriate anonymity.

#### 5.1.3 Off-line password guessing attack

Suppose if A can steal the $REQUEST\{M_{1},M_{2}\}$ but password is secured in *M*_2_ and for retrieving the password from *M*_2_ it is required to calculate the *PWD*_*i*_ and it is impossible for A to obtain these parameters due to the security of hash function. Even if the password is compromised, it is impossible for A to prove the legitimacy of the password. Hence, the present protocol withstands against off-line password guessing attack.

#### 5.1.4 Mutual authentication

In the present protocol both user $U_{i}$ as well as server S compute $Auth_{s}^{{}^{\prime }}=h(sk_{s}\|PWD_{i}^{{}^{\prime }}\left\|D\right)$ and verifies $Auth_{s}^{{}^{\prime }}\overset{?}{=}Auth_{ui}$ on server side and similarity, $Auth_{s}^{{}^{\prime }}=h(sk_{ui}\|PWD_{i}\left\|X\right)\overset{?}{=}Auth_{s}$ on client side. So, improved scheme fulfills the requirement of mutual authentication.

#### 5.1.5 Perfect forward secrecy

Suppose if the secret keys of $U_{i}$ and S are compromised, A is still unable to guess the session keys *sk* = *r*_1_.*r*_2_.*P*. It is infeasible for A to compute *r*_1_ and *r*_2_ from *X* and *D*, respectively, due to ECDLP. Hence, the present protocol provides forward secrecy.

#### 5.1.6 Masquerading attack

For user masquerading, A requires user password *PW*_*i*_ to compute the valid value of *PWD*_*i*_. Assume A successfully intercepts the request message {*M*_1_, *M*_2_}, it is not possible to compute the value of *PWD*_*i*_ from *M*_1_, *M*_2_ due to the hardness of ECDLP and *M*_2_ contains the encrypted value by *X*. For server masquerading attack, A requires verifier *V PW* from server database and secret key $P_{s}$, which is only known to the server. Without $P_{s}$ server secret key, A is unable to compute the *PWD*_*i*_ from *V PW*. For authenticating the user, A also needs the *r*_1_ to compute the *X*, so, A is unable to authenticate *E*_*X*_(*t*_*i*_‖*ID*_*i*_‖*X*‖*PWD*_*i*_) = *M*_2_. Moreover, *r*_2_ is required to compute *sk*_*s*_ and *Auth*_*s*_. Hence, the proposed scheme resists against masquerading attack.

#### 5.1.7 Resist insider attack

In registration phase of the newly stated protocol. $U_{i}$ transmits a message containing (*ID*_*i*_, *PWD*_*i*_) instead of (*ID*_*i*_, *PW*_*i*_), where $PWD_{i}=h(ID_{i}\|PW_{i}\left\|P_{ui}\right)$. So, A is incapable of obtaining the user’s password *PW*_*i*_ without knowledge of $P_{ui}$. Hence, it is impossible for A to launch the insider attack.

#### 5.1.8 Session key secrecy

The two random numbers *r*_1_ and *r*_2_ are used to compute the session key for every session. These two numbers are chosen by the server S and user $U_{i}$, independently, which is different in each session. So, if one of the session key is exposed, the rest of the session keys will persist. Hence, the new proposal achieved the session key secrecy.

### 5.2 Formal security analysis

This segment, demonstrates the formal security analysis of the present protocol. This analysis validates the claim about the proposed scheme that it is provably secured.

**Theorem 1** *The robust authentication protocol with anonymity using ECC for session initiation protocol PREBMAPSIP is provably secured against an adversary* A. *Due to ECDLP assumption and security of hash function*, *it is impossible to obtain the user* $U_{i}$’*s* *real identity* *ID*_*i*_, *password* *PW*_*i*_, *user private key* $P_{ui}$ *and shared session key* *sk* *within user* $U_{i}$ *and the server* S.

**Proof 1** *For analysis of the present stated protocol that it is provably secured*, *the similar model as* [33, 34, 36, 37] *is adopted*.

*Before proceeding ahead*, *following oracles are defined*:

- **Extract 1**: *This oracle results from input A out of a secure one-way hash function B* = *h*(*A*).
- **Extract 2**: *Result of this oracle is the plain text p from cipher text C* = *Ek(p) without the knowledge of shared symmetric key k*.
- **Extract 3**: *This oracle returns the integer multiplier* *a* *out of given an ECC point* *a*.*P*.

A *performed the experiment* $exp1_{A,PREBMAPSIP}^{Hash,Ecdlp,Symenc}$ *to break the proposed* *PREBMAPSIP* *protocol*. A *is allowed to use* Extract 1, Extract 2 *and* Extract 3 *oracles for the said purpose*. A *has the potential to retrieve the user’s private key* $P_{ui}$, *user’s password* *PW*_*i*_ *and shared session key* *sk* *by executing the experiment* $exp1_{A,PREBMAPSIP}^{Hash,Ecdlp,Symenc}$ *and by use of oracles* Extract 1, Extract 2 *and* Extract 3. *The probability of success for the said experiment* $Adv1_{A,PREBMAPSIP}^{Hash,Ecdlp}\left(t_{exc},q_{r1},q_{r2},q_{r3}\right)=maximum_{A}\left(succ_{1}\right)$, *where*, A *performed several queries* *q*_*re*1_, *q*_*re*2_ *and* *q*_*re*3_ *in polynomial time* *t*. *According to experiment* $exp1_{A,PREBMAPSIP}^{Hash,Ecdlp,Symenc}$, A *can break the* *PREBMAPSIP* *security if and only if he can (1) break security of One-way hash function (inversion)*, *(2) obtained the plain text without the knowledge of key (Decipher text) and (2) break* *ECDLP (extracting scalar)*. *However*, *it is computationally impractical to break One-way hash function*, *decrypt the message without key and ECDLP*. *Hence*, *the newly presented protocol for SIP is provably secure against* A *to acquire* *ID*_*i*_, *PW*_*i*_, $P_{ui}$, *and* *sk*.

**Algorithm 1** $exp1_{A,PREBMAPSIP}^{Hash,Ecdlp,Symenc}$

1: Eavesdrop the request message (*M*_1_, *M*_2_), Where *M*_2_ = *E*_*X*_(*t*_*i*_‖*ID*_*i*_‖*X*‖*PWD*_*i*_)

2: Call Extract 2 on *E*_*X*_(*t*_*i*_‖*ID*_*i*_‖*X*‖*PWD*_*i*_) to obtain $\left(t_{i}^{{}^{\prime }}\|ID_{i}^{{}^{\prime }}\|X^{{}^{\prime }}\|PWD_{i}^{{}^{\prime }}\right)$ ← *Extract* 2 (*M*_2_).

3: Call Extract 3 on (*X*′) to obtain $\left(r_{1}^{\prime }\right)$ ← *Extract* (*X*′).

4: Eavesdrop challenge message (*M*_3_, *Auth*_*s*_), Where *Auth*_*s*_ = *h*(*sk*_*s*_‖*T*‖*X*)

5: Call Extract 1 on *h*(*sk*_*s*_‖*T*‖*X*) and get $\left(sk_{s}^{\prime },T^{\prime \prime },X^{\prime \prime }\right)$ ← *Extract* 1 (*Auth*_*s*_)

6: Call Extract 3 on (*X*″) to obtain $\left(r_{1}^{\prime \prime }\right)$ ←*Extract* (*X*″).

7: then if $\left(r_{1}^{\prime }=r_{1}^{\prime \prime }\right)$.

8: Compute $X^{\prime \prime \prime }=r_{1}^{\prime }.P$.

9: **if** *X*′ = *X*‴ **then**.

10:  Accept $ID_{i}^{\prime }$

11:  Call Extract on *PWD_i_* to obtain ($ID_{i}^{\prime \prime }$, $PW_{i}^{\prime }$, $P_{ui}^{\prime }$) ← *Extract* 1 (*PWD_i_*).

12:  Accept the deduced $ID_{i}^{\prime \prime }$, $PW_{i}^{\prime }$, $P_{ui}^{\prime }$ and $sk_{s}^{\prime }$ as the appropriate user’s identity *ID_i_* user’s password *PW_i_*, user’s secret key $P_{ui}$ and session key *sk_s_* between $U_{i}$ and S.

13:  **return** Success

14: **else**

15:  **return** Fail

16: **end if**

## 6 Performance & security comparisons

### 6.1 Computation cost analysis

The present protocol’s performance and security analysis is evaluated with previously stated schemes [24–29, 38–41] in this segment. Registration phase is performed only once before authentication, so, the authentication phase mainly focuses on the performance comparison. For performance calculation, the notation used for the different cryptographic operation are as follows:

- *t*_*sh*_: time to compute secure One-way hash function
- *t*_*em*_: time to calculate point multiplication
- *t*_*ea*_: time to calculate point addition operations
- *t*_*emt*_: time to calculate map to point operation
- $t_{E_{s}}$: time for a symmetric encryption/decryption

The running times for *t*_*sh*_, *t*_*em*_, *t*_*ea*_, *t*_*emt*_ and $t_{E_{s}}$ are approximately 0.0023 ms, 2.226 ms, .0288 ms, 0.947 ms and 0.0046 ms, respectively mentioned by Kilinc and Yanik [42] recently. Furthermore, XOR and inverse operation are neglected due to the insignificance of these operation, as indicated by Kilinc and Yanik. Performance comparison is demonstrated in the Table 2 with the recent allied schemes.

**Table 2:** Computational cost analysis.

Schemes	User	Server	Total	Running time
Lu et al. [29]	4*t*_*sh*_ + 2*t*_*em*_	4*t*_*sh*_ + 2*t*_*em*_	8*t*_*sh*_ + 4*t*_*em*_	≈ 8.9224
Arshad et al. [24]	4*t*_*sh*_ + 2*t*_*em*_	4*t*_*sh*_ + 3*t*_*em*_	8*t*_*sh*_ + 5*t*_*em*_	≈ 11.1484
Yoon et al. [25]	2*t*_*sh*_ + 2*t*_*em*_ + 1*t*_*ea*_	2*t*_*sh*_ + 4*t*_*em*_ + 2*t*_*ea*_	4*t*_*sh*_ + 6*t*_*em*_ + 3*t*_*ea*_	≈ 9.4892
Xie’s. [26]	3*t*_*sh*_ + 3*t*_*em*_ + 1*t*_*ea*_	2*t*_*sh*_ + 3*t*_*em*_ + 1*t*_*Es*_	5*t*_*sh*_ + 6*t*_*em*_ + 1*t*_*ea*_ + 1*t*_*ES*_	≈ 13.4009
He et al. [38]	3*t*_*sh*_ + 3*t*_*em*_	3*t*_*sh*_ + 3*t*_*em*_	6*t*_*sh*_ + 6*t*_*em*_	≈ 13.3698
Farash et al. [27]	4*t*_*sh*_ + 2*t*_*em*_	3*t*_*sh*_ + 2*t*_*em*_	7*t*_*sh*_ + 4*t*_*em*_	≈ 8.9201
Zhang et al. [39]	6*t*_*sh*_ + 3*t*_*e*_ + 1*t*_*ea*_	3*t*_*sh*_ + 3*t*_*em*_ + 2*t*_*ea*_	9*t*_*sh*_ + 6*t*_*pm*_ + 3*t*_*ea*_	≈ 13.4631
Yeh et al. [40]	7*t*_*sh*_ + 4*t*_*em*_ + 2*t*_*ea*_	6*t*_*sh*_ + 4*t*_*em*_ + 2*t*_*ea*_ + 1*t*_*emt*_	13*t*_*sh*_ + 8*t*_*em*_ + 4*t*_*ea*_ + 1*t*_*emt*_	≈ 18.9001
Zhang et al. [28]	4*t*_*sh*_ + 3*t*_*em*_	5*t*_*sh*_ + 3*t*_*em*_	9*t*_*sh*_ + 6*t*_*em*_	≈ 13.3767
Tu et al. [41]	7*t*_*sh*_ + 3*t*_*e*_ + 1*t*_*ea*_	3*t*_*sh*_ + 4*t*_*e*_ + 2*t*_*ea*_	10*t*_*sh*_ + 7*t*_*e*_ + 3*t*_*ea*_	≈ 15.6914
Proposed	3*t*_*sh*_ + 2*t*_*em*_	4*t*_*sh*_ + 2*t*_*em*_	7*t*_*sh*_ + 4*t*_*em*_	≈ 8.9247

Table 2 demonstrates that the present protocol possess the same running time as compared with the previously proposed schemes [27]. Moreover, [27] is found to be insecure against some known attacks. The remaining schemes [24–26, 28, 29, 38–41] almost have relatively extended running time and they are also found insecure against some known attacks. Moreover, proposed scheme is provably secure and resists against all possible attacks shown in section 5.

### 6.2 Communication cost analysis

The communication cost analysis is demonstrated in Table 3 of the present protocol with the counterpart schemes [24–29, 38–41]. The present protocol acquires same or less communication overhead as compared with relevant schemes [24–26, 28, 29, 38–41], whereas, it has some additional communication overhead as compared to [27]. However, in term of the number of messages exchanged, the present protocol provides better performance as compared to previously stated schemes.

**Table 3:** Communication cost analysis.

Schemes →	Proposed	[29]	[24]	[25]	[26]	[38]	[27]	[39]	[40]	[28]	[41]
Communication overhead(Bits)	800	960	960	1120	1120	960	640	1120	1440	800	960
Exchanged Messages	3	3	3	3	3	3	2	3	5	3	3

### 6.3 Security comparison

In Table 4, the comparison of security parameters for the present protocol with conventional schemes [24–29, 38–41] is summarized. It is easier to draw the conclusion from Table 4 that the proposed scheme results are better as compared to its counterpart other conventional schemes. The proposed scheme not only outrun in efficiency but also provides mutual authentication. The proposed protocol is robust against the user as well as server masquerading attack.

**Table 4:** Comparison of security parameters.

Schemes:	Proposed	[29]	[24]	[25]	[26]	[38]	[27]	[39]	[40]	[28]	[41]
Resist Replay Attack	✔	✔	✔	✔	✔	✔	✘	✘	✘	✘	✘
Provide Anonymity and Privacy	✔	✘	✘	✘	✘	✘	✘	✘	✔	✔	✘
Resist Off-line Password guessing Attack	✔	✔	✘	✘	✔	✔	✔	✔	✔	✔	✔
Provide Mutual Authentication	✔	✔	✔	✔	✔	✔	✔	✔	✘	✔	✔
Provide Forward Secrecy	✔	✔	✔	✔	✔	✔	✔	✔	✔	✔	✘
Resist User Masquerading Attack	✔	✘	✘	✔	✔	✔	✔	✘	✔	✔	✔
Resist Server Masquerading Attack	✔	✔	✔	✔	✔	✔	✔	✔	✔	✔	✔
Resist Insider Attack	✔	✘	✔	✔	✔	✔	✔	✘	✔	✔	✘
Provide Session Key Secrecy	✔	✔	✘	✘	✘	✘	✔	✘	✔	✘	✔
Resist Stolen-Verifier Attack	✔	✔	✔	✘	✔	✔	✔	✔	✔	✔	✔
Incorrectness	✘	✔	✘	✘	✘	✘	✘	✘	✘	✘	✘
Resist Man In Middle Attack	✔	✔	✔	✘	✔	✔	✔	✘	✔	✔	✘

✔ = Yes, ✘ = No

## 7 Conclusion

In this research work, the Lu et al.’s scheme is cryptanalyzed and it is exhibited that the protocol is insecure against the server and user masquerading attacks. Moreover, the login and authentication phase is found to be incorrect. To overcome these drawbacks, a novel technique is proposed for reducing the processing time and enhancing the system protection. It is proved to be relatively more secure than the conventional techniques as it is verified through well known random oracle model. Hence, the proposed technique provides enhanced security and better performance. So, it is suitable for the practical applications.
